# Matlab algorithms for traffic light assignment using fuzzy graph, fuzzy chromatic number, and fuzzy inference system

**DOI:** 10.1016/j.mex.2020.101136

**Published:** 2020-11-25

**Authors:** Isnaini Rosyida, Nurhaida, Alfa Narendra, Widodo

**Affiliations:** a Universitas Negeri Semarang, Indonesia; b Universitas Papua, Indonesia; c Universitas Gadjah Mada, Indonesia

**Keywords:** Fuzzy graph, Fuzzy chromatic number (FCN), Phase, Mamdani-FIS, Traffic light, Matlab

## Abstract

We propose algorithms in Matlab that combine fuzzy graph, fuzzy chromatic number (FCN), and fuzzy inference system (FIS) to create traffic light assignment based on traffic flow, conflict, and queue length in an intersection. We evaluate the algorithms through two case studies each on a signalized intersection at Semarang City (Indonesia) and compare the result to the existing systems. The case studies show that the algorithm based on fuzzy graph-FCN-FIS could reduce traffic light cycle time on the intersections.

We provide three results as follows:

• A pseudocode to construct fuzzy graph of traffic data in an intersection.
• Algorithm 1 is to Determine fuzzy graph model of a traffic light data and phase scheduling using FCN function which is presented using Matlab programming language.
• Algorithm 2 is to Determine duration of green lights of each phase using Mamdani-FIS codes in Matlab.

## Graphical abstract

**Figure:**
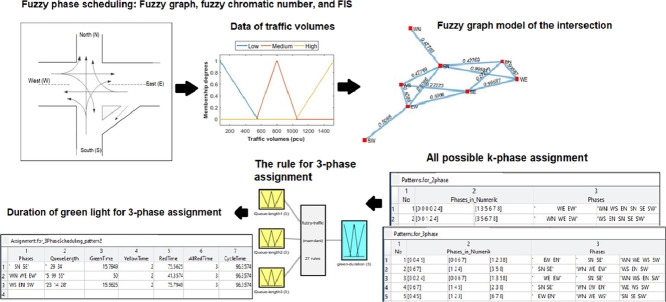

Specifications table

**Table:** 

Subject Area	Computer Science, Mathematics
More specific subject area	Fuzzy systems, computational methods of a fuzzy graph and its chromatic number for traffic light assignment.
Method name	Fuzzy graph and FCN for traffic light assignment.
Name and reference of original method	I. Rosyida, Widodo, C. R. Indrati, and K. A. Sugeng, “A new approach for determining fuzzy chromatic number of fuzzy graph,” J. Intell. Fuzzy Syst., 28(5), 2331–2341, 2015.
	I. Rosyida, Widodo, C. R. Indrati, D. Indriati, and Nurhaida, “Fuzzy chromatic number of union of fuzzy graphs: An algorithm, properties and its application,” Fuzzy Sets Syst., 384, 115–131, 2020.
Resource availability	*–*

## Method details

Throughout this paper, we use some terms in traffic light problems. A phase on an intersection is a portion of a signal cycle when the green lights are assigned to a certain combination of traffic movements. Traffic flow is the study of the movement of individual drivers and conveyances among two locations and the interactions between them. Meanwhile, a volume is defined as a number of traffic elements flowing an area on a road per unit of time (vehicles/hour or passenger car unit/hour) [1]. One of the most important problems that is needed to be solved is traffic congestion. In Indonesia, generally, a fixed phase and fixed time period of green lights on each phase are common applied on an intersection. In this case, whether there are or none vehicles passing through the intersection, the green light is still on until the time elapses. This condition will lead to accumulating queue on a lane with a high traffic volume, particularly at peak hour. One of the alternative options is to create a traffic assignment where the phase scheduling applied considers traffic flow, conflict, and queue. We call this state as a fuzzy phase scheduling.

In general, we model traffic flow on an intersection by using classical graph G(V,E) where V a vertex set and E an edge set, represent a set of traffic flows and a set of conflicting traffic flows, respectively. Two vertices that are connected by an edge mean that the flows are in conflict and should be assigned in different phases. In modeling traffic flow using graph vertex coloring, a color represents a phase on an intersection. A minimum number of colors used in the vertex coloring of G, called as chromatic number, represents a minimum number of phases required on the intersection. Given the load of traffic volumes on conflicting flows, it is interesting to figure out safety level on these flows. Moreover, a conflict between two traffic flows cannot be predicted exactly because it highly depends on their traffic volumes which vary and fluctuate in time. In other words, magnitudes of the conflict exhibit fuzzy phenomena. Hence, we need an alternative graph that accommodates indeterminate phenomena on conflicting traffic flows to model traffic light assignments. Therefore, we proceed to model traffic flows in an intersection in a fuzzy graph where, on each edge, we assign a degree of membership that represents a degree of conflict between two vertices. Next, by using fuzzy chromatic number (FCN) of the graph we produce the number of phases, their respective degree of safety and their phase scheduling. We choose, heuristically a set of phase schedulings to be implemented in an intersection. By setting queue length of flows in a phase scheduling as the inputs and duration of green light as the output of a Mamdami-FIS, we are able to get the desire traffic assignment which the green light setting considering the queue length of the respective flows. This assignment, as can be seen later, has shorter cycletime compared to fixed green duration setting. Further, we use term “traffic flows” in place of “traffic movements” and vice versa, both to describe vertices.

In one hand, several researchers studied traffic light problems using fuzzy graph in [2], [3], [4], [5]–6], However, these works studied only on fixed phases and none of these works gave *fuzzy* phase scheduling and computation of their proposed method. In other hand, many researchers applied only fuzzy logic in traffic light problems. The application of fuzzy logic i.e., Fuzzy Inference System (FIS), in traffic control was first initiated by Pappis and Mamdani [7]. Later, many researchers discussed this problem, such as in [8], [9], [10], [11], [12], [13], [14]–15]. In contrast to other research of traffic light based on fuzzy graph or FIS, this research focuses on constructing fuzzy phase scheduling that links fuzzy graph, FCN and FIS.

Different traffic flows on different conditions ideally require different phase scheduling. Hence, it can be said that setting an optimal phase is a fuzzy phenomenon. In this research, we propose a phase scheduling that considers traffic intensities using fuzzy graph and FCN. Firstly, we construct an algorithm in Matlab to model a traffic light system using fuzzy graph. Secondly, we determine the phase scheduling using FCN algorithm and calculate duration of green lights using Mamdani-FIS function in Matlab. The concept and algorithm of FCN were given in the main article [16,17]. Basic terminologies used in the algorithm are presented in the appendix.

The algorithm is divided into two parts as follows:

1. Determining fuzzy graph model of a traffic light system and phase scheduling using FCN function.
2. Determining duration of green lights of the phases in part (1) using Mamdani-FIS codes in Matlab.

## Determining fuzzy graph and phase scheduling

We constrcut a pseudocode to represent traffic data in an intersection into a fuzzy graph as shown in Table 1.

**Table 1:** Pseudocode to construct fuzzy graph of traffic data in an intersection.

Steps	Commands
1	**Input**V={v_1_,v_2_,…, v_nv_}% vertices/movements
2	**Input**FV={fv_1_,fv_2_,…,fv_nv_}% weight of vertices/load of movements
3	**Input**E={e_1_,e_2_,…,e_ne_|e=(v_i,_v_j_),i≠j,i,j = 1,…,nv}% edges/conflicting movements
4	**Count**maks=maximum of FV
5	**Count**mins=minimum of FV
6	**Count**width=round((maks-mins+4)/3)
7	**Count**L1=mins-2+width
8	**Count**L2=L1–20
9	**Count**L4=maks+2-width
10	**Count**L3=L4+20
11	**Count**L=(L4+L1)/2
12	**Set**μ_low_=trapezoidal(0,0,mins-2,L1)
13	**Set**μ_medium_=triangular(L2,L,L3)
14	**Set**μ_high_=trapezoidal(L4,maks+2,maks+10, maks+10)
15	**Count**FE={fe_1_,fe_2_,…,fe_ne_|fe=max{fv_i,_fv_j_}}
16	**For**k = 1 to ne
17	**If**mins-2 ≤ fe_k_< L1
18	W_k_=μ_low_(fe_k_)
19	**Elseif**L2 ≤ fe_k_< L3
20	W_k_=μ_medium_(fe_k_)
21	**Else**
22	W_k_=μ_high_(fe_k_)
23	**EndIf**
24	μ_E_(fe_k_)=W_k_
25	**EndFor**
26	**Set**$\tilde{E}=(E,\mu_{E})$ **%**fuzzy edge set
27	**Set**$\tilde{G}=\left(V,\tilde{E}\right)$ **%**fuzzy graph G

Data of traffic are first inputed into a mat file. The following scripts are used.



As shown on the above script, we use an intersection with 4 approaches, namely: W=west, N=north, E=east, and S=south. If users want to applied this program in an intersection with more than 4 approaches, than they can modify these commands. There are 4 inputs. Flows is a set of traffic flows which are represented as vertices in the fuzzy graph. Flow `WN'states that there is a traffic movement from West to North. Users should identify which traffic flows represents the intersection and they can modify this step based on traffic assignments in the intersection. Volumes is a set of the number of vehicles for all traffic flows. The third input is Conflicts. It is a set of conflicting traffic flows. They are represented as edges in the fuzzy graph. For example, let traffic flow `WN’ and SN' are in conflict. Then, there is an edge `WN SN' in the edge set. Lastly, we input Queue, a set of queue length of the flows, Flows. This input, will be used later in the next part. The users can modify this step according to conflicting traffic flows that they are observed in the intersection. Step 1 in the script is the first 3 steps on the pseudocode. The following command on Matlab command window shows the load of mat.file ‘case1.mat’ that we input on the above algorithm.



Further, we create the following algorithm which are based on the above pseudocode and FCN in [17]. We call this algorithm as Algorithm 1 for ease of referencing in this article.

**Table:** 

**Algorithm 1**

Explanation of Algorithm 1 is as follows:

In step 1. We load traffic data called case1.
In step 2, fuzzy graph construction is done. We calculate crisp volume of conflicting movements or crisp weight of edges in step 2.1. In step 2.2 and step 2.3, we determine intervals and fuzzy sets of traffic volumes, i.e., {(low,μ_low_), (medium,μ_medium_), (high,μ_high_)} where μ is the membership function and we display the membership functions of the fuzzy sets, respectively. We fuzzify the edge crisp weights in step 2.4 to get the fuzzy edge set, $\tilde{E}=(E,\mu_{E})$. Plot of the fuzzy graph is given in step 2.5. The script on this step describes step 4 up to the last step in the pseudocode.

Determining the phase scheduling is done in step 3. We use a call function namely FCNwithpartition which is on the appendix B. This function is a slighty modified form of its original flowchart in [17]. Input for this function is a fuzzy edge set, while outputs are k,Lk and P. The output k is chromatic number. In graph traffic modeling, it represents the number of phases. Lk is the degree of safety of its respective value k. Setting both side by side, we get (k,Lk) is the fuzzy chromatic number. The output P is the associated phase scheduling to the number of phases k. Also, known as k-phase scheduling. Saving the phase scheduling in a mat file to be processed further as input for the second part of the modeling is done in the last step, step 4.

The result of Algorithm 1 is a mat file called, in this example, 'case1_FuzzyPhases.mat'. The following scripts on Matlab Command Window show the content of the file. There are three commands on the window. The first command is to load  the mat file. The file contains one structure namely Patterns. The second command is to check the contents of Patterns. There are four substructures on Patterns. The last command, Patterns. FuzzyChromaticNumber displays FCN, i.e., the number of minimum phases, k and its respective degree of safety, Lk.



The next step is to determine phases which are eligible for fuzzy phase scheduling. Given that FCN shows safety level of the number of phases applied on the traffic network, to be eligible, we can determine the k-phase based on the degree. We omit the case of *k* = 1, i.e. 1-phase scheduling, as definitely not safe (see the above scripts). Meanwhile, arrangement with 3 to 8 phases have 1 safety level. However, as the number of phases applied on an intersection affects the length of cycle time, of all the number of phases which has 1 safety degree we choose the smallest number. In this example, we choose *k* = 3. By that setting we work only on *k* = 2 and *k* = 3 phases. The last 2 substructures on Patterns, i.e., for_3phase and for_2phase, show all possible patterns of their k-phase.

As mentioned previously, chromatic number (k) represents the number of phases in a traffic assignment. A term k-phase scheduling means we assign k phases for traffic assignment on an intersection. Fuzzy chromatic number (k,Lk) gives a degree of applying k-phase scheduling in a traffic assignment. As shown on FCN on the above scripts, 1-phase scheduling comes with the lowest degree (0.0041), as it is obviously not safe if traffic elements are allowed to flow (from all directions) all at once. Assignments within 2 phases comes with higher degree which is 0.5723. Whereas, assignments within 3 or more phases has 1 degree of safety. However, the more the number of phases the lengthier the traffic cycle time. There is inevitably a trade-off between safety level and cycle time length. Therefore, we are looking for a phase scheduling which is safe at a reasonable cycle time.

### Determining duration of green light

Having constructing traffic phases, we would like to determine duration of the traffic lights which considers queue length of flows on the phases. This is done by Mamdami-FIS codes. The highligth of the system is as follows:

1. The input parameter is flowing queue length in each phase and is labelled as Short, Medium, Long. The formula used to determine the queue length in each traffic flow is presented in Eqn 1:
  (1) $$\text{Ql}=\frac{\sum_{i=1}^{3}\left(L_{i}\times Q_{i}\right)}{c\times \text{width}}\;$$

where Ql is the queue length (meter), $L_{i}$ is the area of each vehicle-i (m^2^), $Q_{i}$ is the number of vehicles per hour, c is the number of cycles of green light per hour, width is the wide of the approach (meter), and i is the type of vehicle (i=1 for MC(Motor Cycle), i=2 for LV(Light Vehicle), and i=3 for HV(Heavy Vehicle)).

2. The output parameter is green light-duration in each phase (Short, Medium, Long). According to standard cycle time for the 3-phase or 4-phase scheduling [1], we use the maximum length of interval for the output is 100 s. The fuzzy sets of input and output parameters have triangular membership functions.
3. The fuzzy rules are defined based on the number of phases used and maximum traffic flows in a phase.
4. The defuzzification method is centroid.

The following matlab algorithm which we call Algorithm 2 is mainly composed of Mamdani-FIS in codes.

**Table:** 

**Algorithm 2**

Algorithm 2 consists of three main steps. The first step is loading data. We need to input Queue which is in file case1.mat. The queue length of the movements is calculated using the formula in [1]. We load fuzzy phases obtained in the previous algorithm that is in variable Patterns.allphases in case2_FuzzyPhases mat file. As our Mamdami input is queue length of flows, we need to fuzzify the queue length into fuzzy sets, i.e., short, medium and long where each has triangular membership function. Hence, we need to load the range inRange and parameters of each membership function inMFparams.

Step 2 is the Mamdami-FIS system written in coding. The number of inputs of the FIS is set to be not more than 4, e.g., Queue-length1, Queue-length2, Queue-length3, and Queue-length4. The label Queue-length1 means the queue length in the first flow and so on. Given any k-phase scheduling, Algorithm 2 will work only if maximum number of flows/movements on the phase scheduling is 2,3 or 4. We limit the algorithm due to the empirical fact that applying a phase with more than 4 flows/movements results in a lengthier cycletime.

**Fig. 1:**
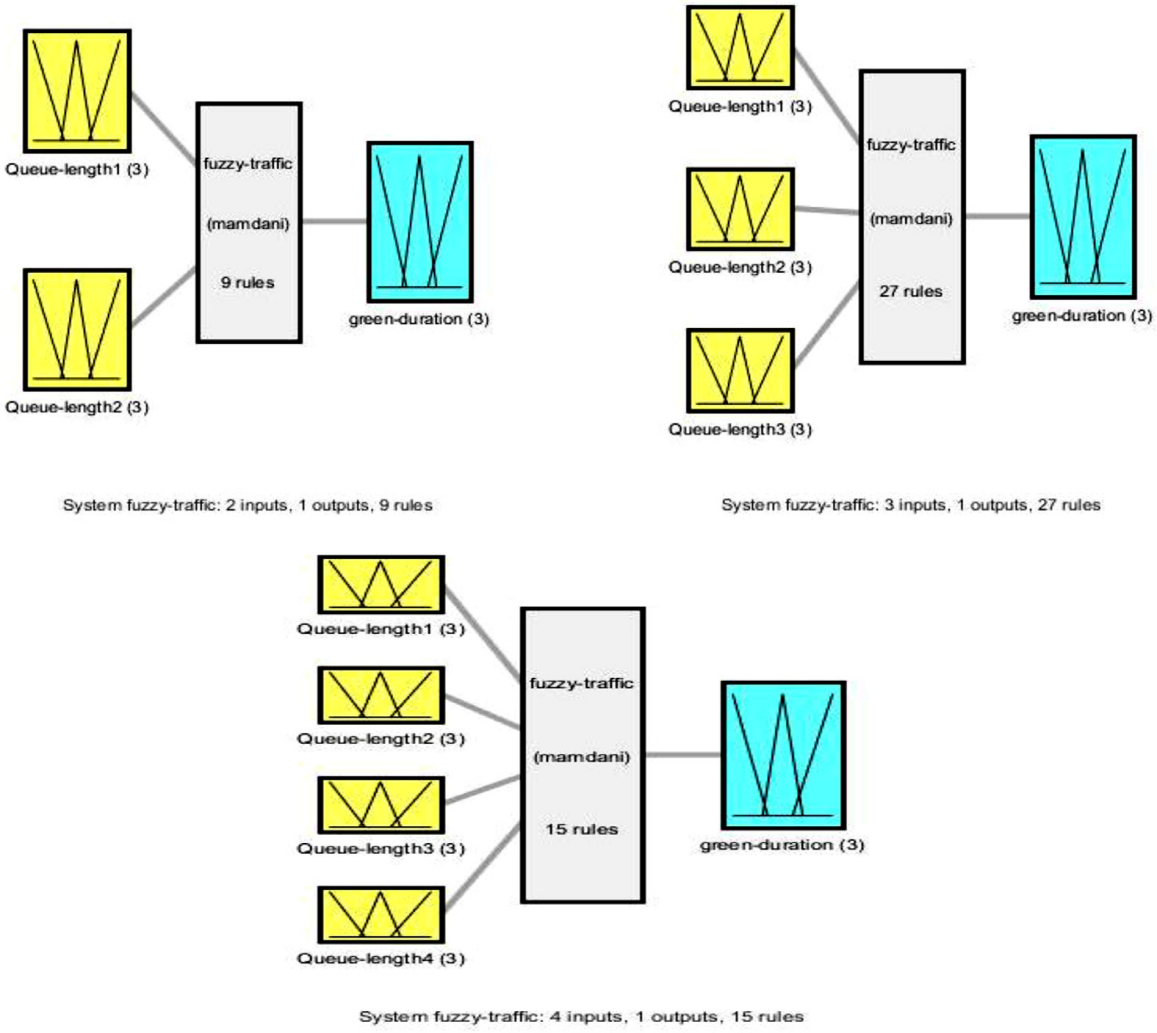
`Fuzzy-traffic'. A Mamdami FIS for k-fuzzy phase scheduling.

There is only one output of our FIS that is duration of green light. The fuzzy output may have three fuzzy numbers ,e.g. {'short';'medium';'long'}. We set maximum duration of green light for a phase is 100 s [1] and set fixed parameters for triangular membership function as [0 0 35; 30 50 70; 60 100 100], respectively. We call our Mamdami-FIS as ‘fuzzy-traffic’. Three plots in Fig. 1 show available systems of the FIS stucture, i.e., the input, output and evaluation. As can be seen, we set 9, 27 and 15 rules for 2, 3 and 4 inputs, respectively. One can see that we use a call function getmyrule() as in command rule = getmyrule(numOfinLabels)in Step 2 Algorithm 2. We give this function in Appendix C.

In step 3, we save all the outputs. By inputting fuzzy phases obtained previously, ‘fuzzy-traffic’ system gives one phase scheduling that is a scheduling for 3 phases. The result is in a mat file namely 'case1_3-FuzzyPhaseScheduling.mat' (see the scripts below). There are 5 options of phase patterns to arrange the traffic using 3 phases. We can see the results interactively in Variable Window as well.



## Experimental results

### Location 1 (Kaligarang intersection, Semarang City, Central Java, Indonesia)

**Fig. 2:**
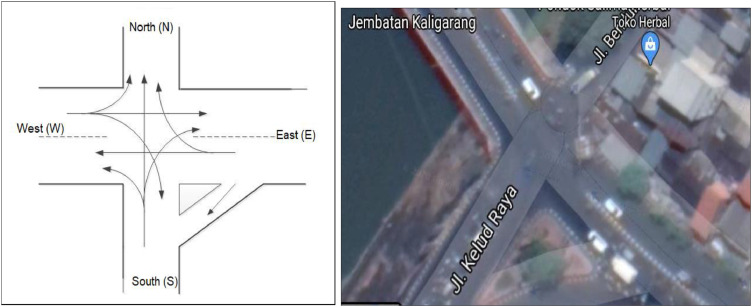
Sketch (left) and image (right) of traffic light system in Kaligarang intersection (Location 1) [18].

Let us consider a case study on an intersection in Semarang City, Central Java, Indonesia, particularly in “Kaligarang” intersection [18]. Fig. 2 shows the sketch and image of the intersection. Data of traffic volumes on all movements are gathered by using video camera during week days (Monday, Tuesday, and Wednesday) on August 6–8, 2018. The data was taken four hours each day e.g., during two peak hours in the morning (06.00–08.00 a.m.) and two peak hours at afternoon (16.00–18.00 p.m.). Data of this example have been shown to illustrate the building of our algorithms (1 and 2).

**Fig. 3:**
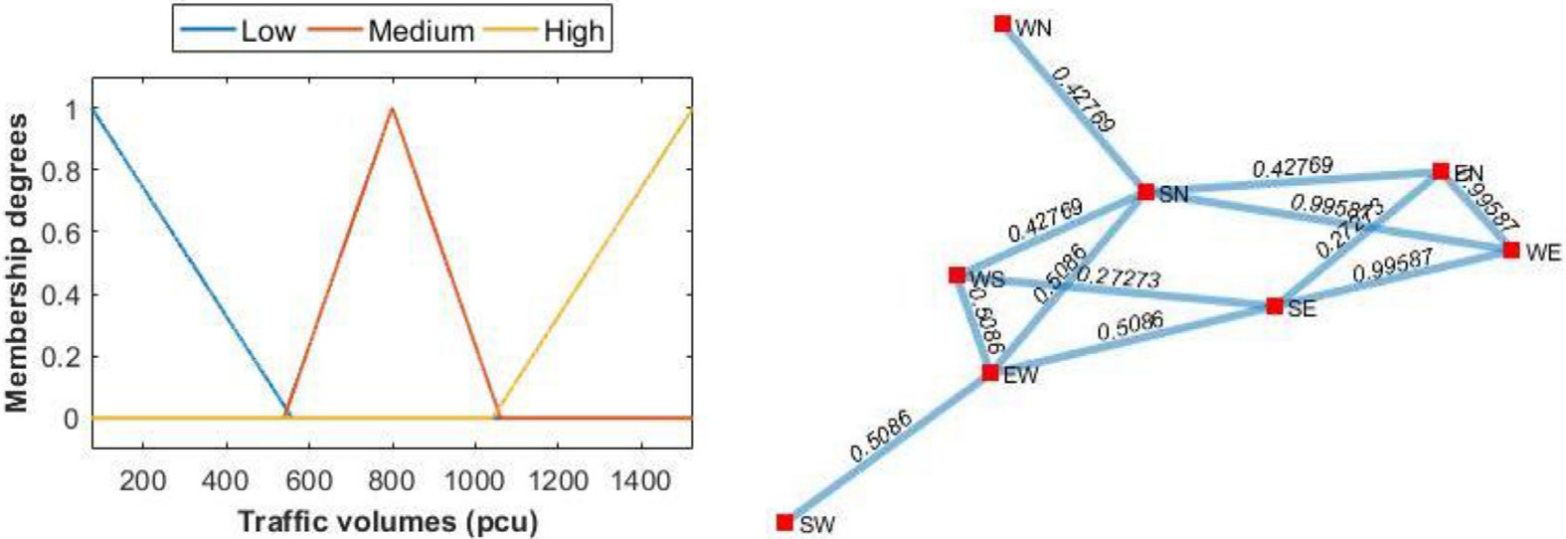
Membership functions of fuzzy edge set in Table 3 (left) and representation of traffic in Fig. 2 into a fuzzy graph (right).

We call this example as case 1. There are four variables to model the intersection into a fuzzy graph. These are traffic flows, traffic volumes, flow queue length and conflicting flows. The first three variables are shown in Table 2 and the fourth variable is on the 2nd or 3rd column of Table 3. Traffic flows and their volumes are used in Algorithm 1. Whereas, queue length is used in Algorithm 2. As said in introduction, in graph modeling, traffic flows and conflicting flows are modeled as vertex (*V*) and edge (*E*) sets, respectively. In our model, we use fuzzy edge set $\tilde{E}=\left(E,W\right)$ that is we assign a degree to each edge in *E*. In doing so, we define membership functions for traffic volumes, i.e., fuzzy sets of traffic volumes. We then construct fuzzy graph of the traffic data, $\tilde{G}=\left(V,\tilde{E}\right)$. Plots of the membership functions and fuzzy graph are shown in Fig. 3. Both plots are the outputs of step 2 in Algorithm 1.

**Table 2:** Traffic flows, volumes and queue length at Location 1 during peak time (morning).

No	Flows	Volumes (pcu)	Queue length (meter)
1	WN	76	5
2	WE	1523	99
3	WS	349	23
4	EW	928	55
5	EN	222	14
6	SN	351	29
7	SE	426	34
8	SW	341	28

**Table 3:** Conflicting traffic flows (edge set), volumes, and their membership degrees

No	Edges (in alphabetical)	Edges (in numerical)		Volumes(pcu)	Membership degrees
1	WN SN	1	6	351	0.4277
2	WE SE	2	7	1523	0.9959
3	WE SN	2	6	1523	0.9959
4	WE EN	2	5	1523	0.9959
5	WS SN	3	6	351	0.4277
6	WS EW	3	4	928	0.5086
7	WS SE	3	7	426	0.2727
8	EW SW	4	8	928	0.5086
9	EW SN	4	6	928	0.5086
10	EW SE	4	7	928	0.5086
11	EN SN	5	6	351	0.4277
12	EN SE	5	7	426	0.2727

As stated previously, output of the first algorithm is fuzzy phases of an intersection. The phases are defined as “fuzzy” because based on FCN definition, a level of safety is assigned to each number of phases. Table 6 shows FCN of traffic network of case 1. The table is interactively screenshootted and cropped from Matlab Variable Window. The number k in Table 4 represents the amount of phases to arrange traffic flows, while Lk represents a possibility to apply the k-phase. According to the table, traffic assignment with 3 number of phases on Location 1 has one degree of safety. Therefore, we can apply *k* = 3 phases during peak time, i.e., between 06.30–09.00 a.m. or between 16.00–18.00 p.m. All possible k-phase scheduling with *k* = 2 and 3 can be seen on Patterns structure (on the Variable Window) as on the screenshots in Table 5.

**Table 4:** Number of phases (k) and the associated degree (Lk) in case 1.

No	Number of phases (k)	Degree of safety (Lk)
1	1	0.0041
2	2	0.5723
3	3	1.0000
4	4	1.0000
5	5	1.0000
6	6	1.0000
7	7	1.0000
8	8	1.0000

**Table 5:** All possible patterns of k-phase for *k* = 2 (top) and *k* = 3 (bottom) in case 1.

No	2-Phase Scheduling in case 1					
	Phase-scheduling (in Numerical)		Phase-scheduling (in Alphabetical)			
1	[2 4]	[1 3 5 6 7 8]	[WE EW]	[WN WS EN SN SE SW]		
2	[1 2 4]	[3 5 6 7 8]	[WN WE EW]	[WS EN SN SE SW]		

No	3-Phase Scheduling in case 1					
	Phase- scheduling (in Numerical)			Phase- scheduling (in Alphabetical)		
1	[4 5]	[6 7]	[1 2 3 8]	[EW EN]	[SN SE]	[WN WE WS SW]
2	[6 7]	[1 2 4]	[3 5 8]	[SN SE]	[WN WE EW]	[WS EN SW]
3	[2 4]	[6 7]	[1 3 5 8]	[WE EW]	[SN SE]	[WN WS EN SW]
4	[6 7]	[1 4 5]	[2 3 8]	[SN SE]	[WN EW EN]	[WE WS SW]
5	[4 5]	[1 2 3]	[6 7 8]	[EW EN]	[WN WE WS]	[SN SE SW]

**Table 6:** The 1st option of scheduling with 2-phase in Table 5.

Phase	Movements / Flows
1	WE, EW
2	WN, WS, EN, SN,SE, SW

Table 5 is comprised of two sub tables. The top and bottom sub tables show options of phases of two and three- phase scheduling, respectively. There are 2 options for 2-phase scheduling whereas 5 alternatives are available for 3-phase scheduling. Table 6 and Table 7 show the first option in 2 and 3-phase scheduling, respectively. As can be seen on these two arrangements, maximum number of movements in 2 phases is 6, whereas maximum number of movements for 3 phases is 4. Later, in the second algorithm which constitutes Mamdami-FIS, we limit the algorithm to proceed only phase schedulings where maximum number of the flows are 2, 3, or 4. This is due to empirical facts that more number of movements/flows in a phase increases the cycle time of the assignment.

**Table 7:** The 1st option of scheduling with 3-phases in Table 5.

Phase	Movements / Flows
1	EW, EN
2	SN, SE
3	WN, WE, WS, SW

The next step is to determine green light duration on each phase. The inputs are fuzzy phases shown in Table 5**.** that is *k* = 3 and *k* = 2 fuzzy phases. The system “fuzzy-traffic” reads these inputs and automatically produces the k-phase scheduling according to the rules created. The name of the output mat file is dynamicaly coding, i.e., the algorithm sets the name based on the number of phases of the phase scheduling that is executed online in the system. Hence, the results may be more than one mat file.

For case 1, we obtain a mat file output named ‘result1_3-FuzzyPhaseScheduling.mat’. It means that the available phase scheduling is only one for this case which is the fuzzy scheduling with 3 phases. There are 5 options of phase patterns suggested in the file. Subtables in Table 8 display duration of traffic lights (in seconds) of their respective scheduling with 3 phases shown on screenshots of Matlab Variable Window. One can see that a phase which has on average higher queue will be assigned longer green time.

**Table 8:** Duration of green, red, and yellow lights of 3-phase scheduling in Location 1 (Morning).

3-Phase Scheduling: Pattern 1							
No	Phases	Queue Length	Green Time	Yellow Time	Red Time	All Red Time	Cycle Time
1	EW EN	55 14	50	2	75.7949	3	130.7949
2	SN SE	29 34	15.7949	2	110	3	130.7949
3	WN WE WS SW	5 99 23 28	50	2	75.7949	3	130.7949
3-Phase Scheduling: Pattern 2							
1	SN SE	29 34	15.7949	2	75.5625	3	96.3574
2	WN WE EW	5 99 55	50	2	41.3574	3	96.3574
3	WS EN SW	23 14 28	15.5625	2	75.7949	3	96.3574
3-Phase Scheduling: Pattern 3							
1	WE EW	99 55	50	2	41.3574	3	96.3574
2	SN SE	29 34	15.7949	2	75.5625	3	96.3574
3	WN WS EN SW	5 23 14 28	15.5625	2	75.7949	3	96.3574
3-Phase Scheduling: Pattern 4							
1	SN SE	29 34	15.7949	2	72.7333	3	93.5282
2	WN EW EN	5 55 14	12.7333	2	75.7949	3	93.5282
3	WE WS SW	99 23 28	50	2	38.5282	3	93.5282
3-Phase Scheduling: Pattern 5							
1	EW EN	55 14	12.7333	2	75.7949	3	93.5282
2	WN WE WS	5 99 23	50	2	38.5282	3	93.5282
3	SN SE SW	29 34 28	15.7949	2	72.7333	3	93.5282

The 5th pattern of phases in the phase scheduling (on the bottom on Table 8) has similar pattern to three phase scheduling in the existing system shown in Table 9. We can see that the proposed fuzzy phase scheduling reduces cycle time of the traffic light system, i.e. cycle time of the proposed phase scheduling, has lower length. Therefore, the proposed method offers a valuable alternative traffic assignment with 3 phases on the intersection.

**Table 9:** Duration of green, red, and yellow lights of the existing system in Location 1 (Morning).

Phases	Queue	Duration (in seconds)				
		Green	Yellow	Red	All-red	Cycle length
{WN,WE,WS}	{5;99;23}	25	2	130	3	160
{SN,SW,SE}	{29;34;28}	70	2	85	3	160
{EW,EN}	{55,14}	50	2	105	3	160

### Location 2 (Lamper Gadjah intersection, Semarang City, Central Java, Indonesia)

**Fig. 4:**
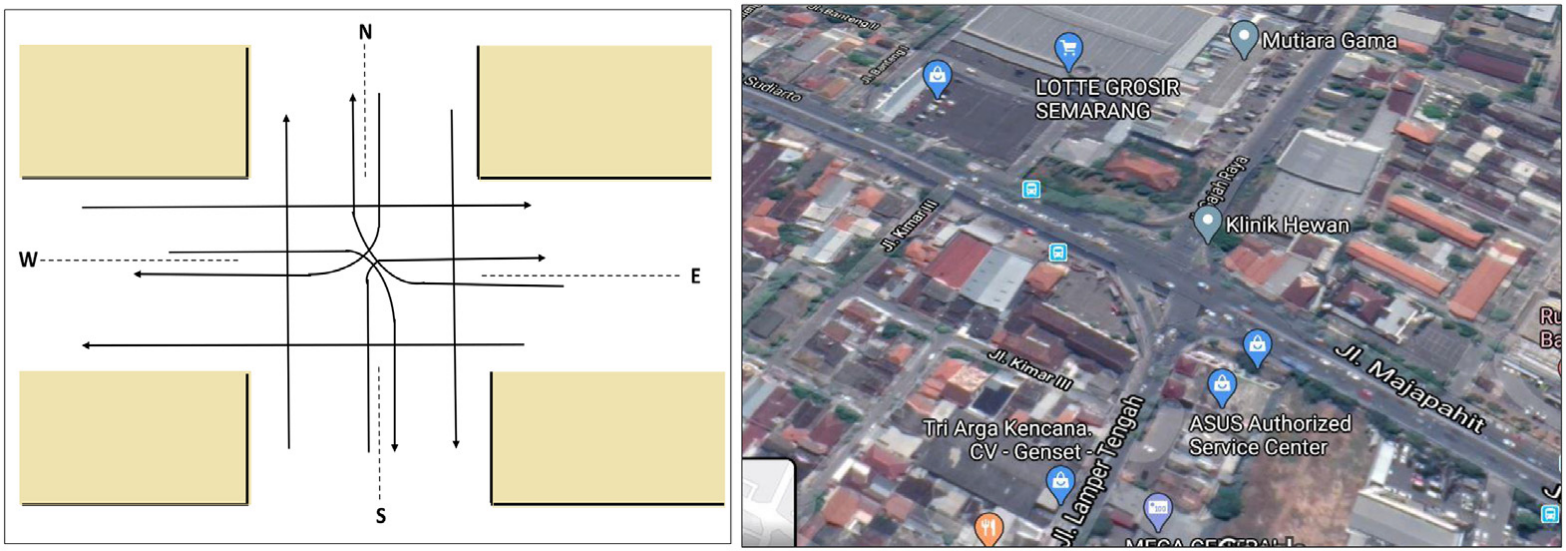
Schema (left) and image (right) of traffic light system in Lamper intersection (Location 2) [19].

**Fig. 5:**
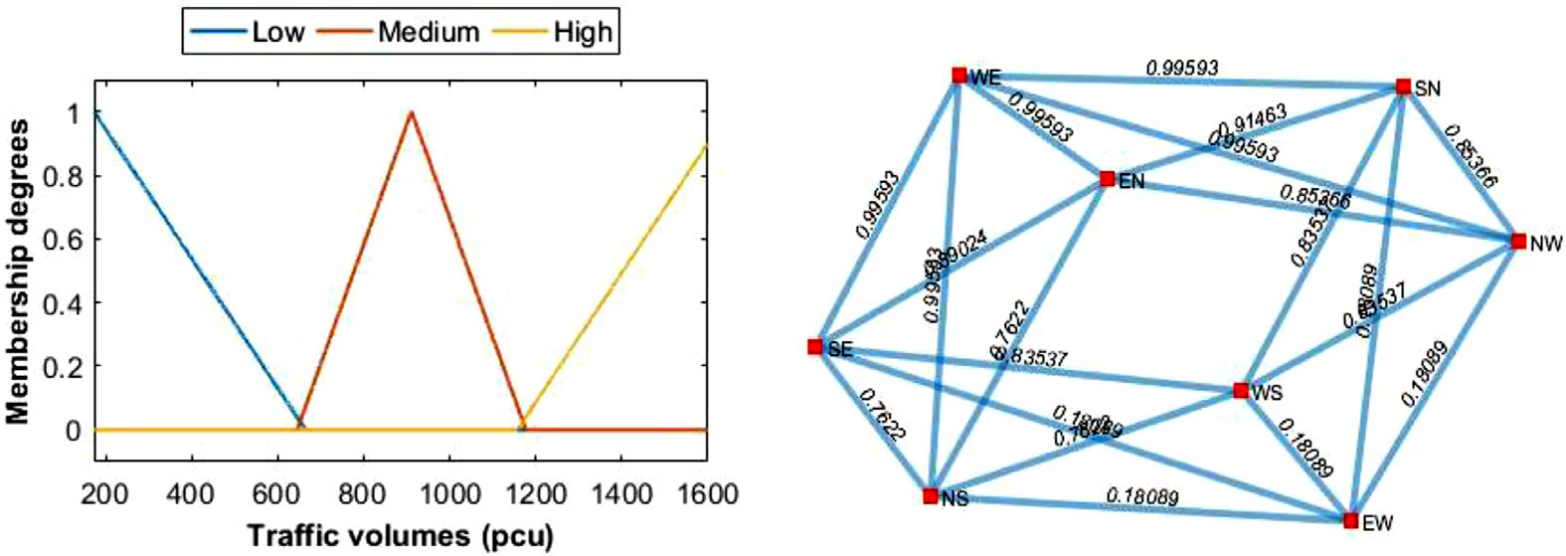
Membership functions of fuzzy edge set (left) and representation of traffic light in Fig. 4 into a fuzzy graph (right).

Let us consider the second case study at Lamper Gadjah intersection in Semarang City, Central Java, Indonesia. Fig. 4. displays the schema and image of Lamper intersection. Data of traffic volumes acquired from video camera during week days (Monday-Friday) on August 2018 [25]. The data was taken on one hour each day e.g., during peak hour in the afternoon (16.00–17.00 p.m.). Data of this example is inputed and saved as ‘case2.mat’. In Table 10 and Table 11, we present traffic flows, volumes, queue length and conflicting flows at peak hour and it membership degrees. Further, Step 2 in Algorithm 1 displays plots of membership functions for the edge set and plot of the fuzzy graph (Fig. 5).

**Table 10:** Traffic flows, volumes, and queue length at location 2 during peak time (afternoon).

No	Traffic flows	Volumes (pcu)	Queue length (meter)
**1**	WE	1647	137
**2**	WS	254	39
**3**	EW	1246	103
**4**	EN	175	30
**5**	SN	215	41
**6**	SE	227	42
**7**	NS	290	35
**8**	NW	245	27

**Table 11:** Conflicting traffic flows (edges), volumes and their membership degrees for case 2.

No	Edges (in alphabetical)	Edges (in numerical)		Volumes	Membership_degrees
1	WE SE	1	6	1647	0.9959
2	WE SN	1	5	1647	0.9959
3	WE EN	1	4	1647	0.9959
4	WS SN	2	5	254	0.8354
5	WS EW	2	3	1246	0.1809
6	WS SE	2	6	254	0.8354
7	EW SN	3	5	1246	0.1809
8	EW SE	3	6	1246	0.1809
9	EN SN	4	5	215	0.9146
10	EN SE	4	6	227	0.8902
11	NW EN	8	4	245	0.8537
12	NW EW	8	3	1246	0.1809
13	NW SN	8	5	245	0.8537
14	NW WS	8	2	254	0.8354
15	NW WE	8	1	1647	0.9959
16	NS EW	7	3	1246	0.1809
17	NS SE	7	6	290	0.7622
18	NS WS	7	2	290	0.7622
19	NS WE	7	1	1647	0.9959
20	NS EN	7	4	290	0.7622

Number of phases and its level of safety, the result of FCN function, for case 2 is shown in Table 12. It is shown that assignments with at least 4 phases have degree of safety 1, whereas assignments with less number of phases, i.e., *k* = 3, 2 and *k* = 1 have very low degree of safety. The function also give all possible phase patterns for *k* = 2,3, and 4 as displayed in Table 13.

**Table 12:** Number of phases (k) and the associated degree (Lk) for case 2.

No	Number of Phases (k)	Degree of safety (Lk)
1	1	0.0041
2	2	0.0854
3	3	0.1463
4	4	1.0000
5	5	1.0000
6	6	1.0000
7	7	1.0000
8	8	1.0000

**Table 13:** All possible k-phase patterns for *k* = 2, 3, and 4 for case 2.

No	2-Phase Scheduling in case 2							
	Phase-scheduling (in Numerical)		Phase-scheduling (in Alphabetical)					
1	[1 2 3]	[4 5 6 7 8]	[WE WS EW]	[EN SN SE NS NW]				
2	[1]	[2 3 4 5 6 7 8]	[WE]	[WS EW EN SN SE NS NW]				

No	3-Phase Scheduling in case 2							
	Phase- scheduling (in Numerical)			Phase- scheduling (in Alphabetical)				
1	[5 6]	[1 2 3]	[4 7 8]	[SN SE]	[WE WS EW]	[EN NS NW]		
2	[1]	[4]	[2 3 5 6 7 8]	[WE]	[EN]	[WS EW SN SE NS NW]		
3	[1]	[5 6]	[2 3 4 7 8]	[WE]	[SN SE]	[WS EW EN NS NW]		

No	4-Phase Scheduling in case 2							
	Phase- scheduling (in Numerical)				Phase- scheduling (in Alphabetical)			
1	[1 2]	[3 4]	[5 6]	[7 8]	[WE WS]	[EW EN]	[SN SE]	[NS NW]
2	[1 3]	[2 4]	[5 6]	[7 8]	[WE EW]	[WS EN]	[SN SE]	[NS NW]
3	[1 2]	[3 4]	[5 7]	[6 8]	[WE WS]	[EW EN]	[SN NS]	[SE NW]

All possible phases in Table 13 are then inputed into the second algorithm. However, we need to put in mind that only scheduling with 4 phases that has 1 degree of safety, the other two have very low degrees of safety (see Table 12). We input the range of input variable as [0 140] and intervals of membership functions of input variabels as in inMFparams=[0 0 50; 45 70 95; 90 140 140] (see Algorithm 2).

Two mat files pop up as the result of algorithm 2 suggesting that there are 2 types of phase scheduling. There is only one option in arranging the traffic light in 3 phases (Table 14), whereas, three options are for scheduling with 4 phases (Table 15). In order to validate the result we collect duration of green lights in the existing system (Location 2) which is presented in Table 16. The system is an intersection where 4-phase scheduling is implemented. The scheduling is equal to the second phase pattern in the fuzzy scheduling with 4 phases (shown on the 2nd subtable from bottom on Table 15). We may argue that the fuzzy phase scheduling proposed is superior in reducing the average time a driver spends his/her time on the intersection as the cycle time has shorter length than the cycle time applied in the existing system (in Table 16).

**Table 14:** Duration of green, red, and yellow lights of 3-phase scheduling in Location 2 (afternoon).

3-Phase Scheduling: Pattern 1							
No	Phases	Queue Length	Green Time	Yellow Time	Red Time	All Red Time	Cycle Time
1	SN SE	41 42	15.8798	2	107.1369	3	128.0167
2	WE WS EW	137 39 103	82.3652	2	40.6514	3	128.0167
3	EN NS NW	30 35 27	14.7717	2	108.2450	3	128.0167

**Table 15:** Duration of green, red, and yellow lights of 4-phase scheduling in Location 2 (afternoon).

4-Phase Scheduling: Pattern 1							
No	Phases	Queue Length	Green Time	Yellow Time	Red Time	All Red Time	Cycle Time
1	WE WS	137 39	50	2	95.6514	3	150.6514
2	EW EN	103 30	50	2	95.6514	3	150.6514
3	SN SE	41 42	15.8798	2	129.7717	3	150.6514
4	NS NW	35 27	14.7717	2	130.8798	3	150.6514

4-Phase Scheduling: Pattern 2							
No	Phases	Queue Length	Green Time	Yellow Time	Red Time	All Red Time	Cycle Time
1	WE EW	137 103	87.7275	2	61.0480	3	148.7755
2	WS EN	39 30	15.3966	2	128.3790	3	148.7755
3	SN SE	41 42	15.8798	2	127.8957	3	148.7755
4	NS NW	35 27	14.7717	2	129.0039	3	148.7755

4-Phase Scheduling: Pattern 3							
No	Phases	Queue Length	Green Time	Yellow Time	Red Time	All Red Time	Cycle Time
1	WE WS	137 39	50	2	96.5980	3	151.5980
2	EW EN	103 30	50	2	96.5980	3	151.5980
3	SN NS	41 35	15.7182	2	130.8798	3	151.5980
4	SE NW	42 27	15.8798	2	130.7182	3	151.5980

**Table 16:** Duration of green, red, and yellow lights in the existing system (Location 2).

Phases	Volumes	Duration (in seconds)				
		Green	Yellow	Red	All-red	Cycle Time
{WE, EW}	{137;103}	30	2	133	3	165
{WS, EN}	{39; 30}	30	2	133	3	165
{SN, SE}	{41; 42}	20	2	138	3	165
{NS. NW}	{35; 27}	65	2	98	3	165

## Conclusions

We proposed algorithms to determine the number of phases and the fuzzy phase scheduling for a traffic light system based on fuzzy graph and fuzzy chromatic number. Further, we determine duration of green lights in each phase by applying Mamdani-FIS. We evaluate the algorithms through two case studies. The results show that the combination of the algorithms and the Mamdani-FIS gives some options of phase scheduling with different cycle times. The phase scheduling proposed in this research increases performances of intersections under study in that the cycle times of the proposed scheduling are shorter than that of the existing systems. In our future research, we will improve the performance of the algorithms and implement it on various types of intersections.

## Appendix A

In this part, we discuss basic theories used in the algorithms, such as terminologies in traffic light systems ([1,^20^,21]), fuzzy graph, and fuzzy chromatic number (FCN) ([16,17]).

### Basic terminologies in traffic light systems

Some terminologies in used in this article are as follows:

1. an approach is an area for entering vehicles in a traffic facility;
2. a **traffic conflict**, is "an observable event which would end in an accident unless one of the involved parties slows down, changes lanes, or accelerates to avoid collision" [1]. Traffic conflicts are defined by their time-to-collision, post-encroachment-time, and angle of conflict parameters as well as the vehicles' position in time and space.” [24]
3. Light Vehicle (LV) is an index for vehicle on four wheels (including passenger cars, oplets, micro buses, pick-ups and micro trucks);
4. Heavy Vehicles (HV) is an index for vehicle with two or three wheels (including buses and truck combinations);
5. Motor Cycles (MC) is an index of motor vehicle with two or three wheels (including motor cycles and 3-wheeled vehicles);
6. Passenger car unit (pcu) is a conversion factor for different vehicle types with regard to their impact on capacity as compared to a passenger car (i.e. for passenger cars and other light vehicles, pcu = 1.0).

### Fuzzy graph and fuzzy chromatic number (FCN)

After a concept of fuzzy set was introduced by Zadeh in 1965 [22], the concept of a fuzzy graph that consists of a crisp vertex set and a fuzzy edge set was initiated by Kaufmann in 1973 [23]. A fuzzy set $\tilde{A}$ on *X* is a set {(a,μ(a))|a∈X} with a membership function μ:X↦[0,1]. Further, the classical sets are named as crisp sets.

Given V which is non-empty set and E⊆V×V. A fuzzy graph $\tilde{G}=\left(V,\tilde{E}\right)$ consists of a vertex set V and $\tilde{E}$ which is a fuzzy edge set with μ:V×V↦[0,1] as its membership function. Thereafter, the graph G=(V,E) is named as a crisp graph. In crisp graphs, a set *A* is called an independent vertex set if each pair of vertices in *A* is independent (not adjacent). The concept is generalized into fuzzy graph as follows. Given δ∈[0,1], a fuzzy independent vertex set (FIVS) of $\tilde{G}=\left(V,\tilde{E}\right)$ is a set I⊆V which satisfies the condition μ(xy)≤δ or each pair x,y∈I. The FIVS I can be denoted as $I^{\delta }$. Moreover, a k-coloring of $\tilde{G}$ could be defined through a partition of V into k- FIVS $\left\{I_{1}^{\delta },\;I_{2}^{\delta },\ldots ,I_{k}^{\delta }\right\}$ such that $I_{i}^{\delta }\cap \;I_{j}^{\delta }=\emptyset$ for all i≠j and $I_{1}^{\delta }\cup \;I_{2}^{\delta }\cup \ldots \cup I_{k}^{\delta }=V$. The minimum k in the k-coloring of $\tilde{G}$ is named δ-chromatic number of $\tilde{G}$, symbolized by $\chi^{\delta }\left(\tilde{G}\;\right)$ [24].

Meanwhile, the concept of FCN is as follows. Given a fuzzy graph $\tilde{G}=\left(V,\tilde{E}\right)$ with n vertices. A FCN of $\tilde{G}$, symbolized by $\tilde{\chi }\left(\tilde{G}\;\right)$, is a fuzzy set $\tilde{\chi }\left(\tilde{G}\;\right)=\left\{\left(k,L_{\tilde{\chi }}\left(k\right)\right)|k=1,2,\ldots ,n\right\}$, where $L_{\tilde{\chi }}\left(k\right)=\max\left\{1-\delta |\chi^{\delta }\left(\tilde{G}\;\right)=k\right\}$ is a membership degree of k in the FCN [17].
